# Cryptosporidiosis Decline after Regulation, England and Wales, 1989–2005

**DOI:** 10.3201/eid1304.060890

**Published:** 2007-04

**Authors:** Iain R. Lake, Gordon Nichols, Graham Bentham, Florence C.D. Harrison, Paul R. Hunter, R. Sari Kovats

**Affiliations:** * University of East Anglia, Norwich, England; † Health Protection Agency, London, England; ‡ London School of Hygiene and Tropical Medicine, London, England

**Keywords:** Cryptosporidiosis, Cryptosporidium, environment and public health, weather, regulation, dispatch

## Abstract

Since new drinking water regulations were implemented in England and Wales in 2000, cryptosporidiosis has been significantly reduced in the first half of the year but not in the second. We estimate an annual reduction in disease of 905 reported cases and ≈6,700 total cases.

Cryptosporidiosis is a common cause of gastroenteritis worldwide. In England and Wales, ≈4,500 cases are reported each year (*1*). In the 1990s, several cryptosporidiosis outbreaks in England and Wales were associated with public drinking water supplies; in 2000, new drinking water regulations were implemented to address this problem. Risk assessments were required at all water treatment plants, and those that did not meet the standards were required to monitor regularly for *Cryptosporidium* spp. Consequently, water companies closed some plants, upgraded others, and paid close attention to the maintenance and operation of their works (*2*). Since these regulations were implemented, a reduction in reported cases of cryptosporidiosis, especially the disappearance of the spring peak, has been reported in northwestern England (*3*). The aim of our research was to quantify the public health impact of the regulations by assessing whether they have led to statistically significant reductions in cryptosporidiosis.

## The Study

All cases of cryptosporidiosis in England and Wales reported to national surveillance from 1989 through 2005 were analyzed; those associated with recent foreign travel were excluded. The average weekly number of cryptosporidiosis cases preregulation (1989–1999) were plotted against the same data postregulation (2000–2005) (Figure). Since the regulations were implemented, fewer cryptosporidiosis cases have occurred in the first half of the year but more in the second. However, as the standard deviation bars on the figure indicate, the number of cases fluctuated from year to year both before and after the regulations. This trend makes it difficult to ascertain whether the changes after regulation are part of the natural interannual variability or represent real changes in incidence. It also makes it difficult to quantify the public health impact of the regulations.

**Figure:**
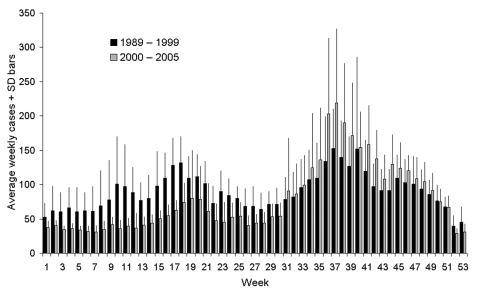
Weekly cryptosporidiosis cases, England and Wales, 1989–1999 and 2000–2005. SD, standard deviation

Climatic variability and community spread from imported travel cases are suggested as the main sources of this interannual variability (*4*,*5*). Precipitation may wash *Cryptosporidium* organisms from land into public water supplies, and warm, dry weather may increase the number of countryside visits. Both of these could result in exposure to *Cryptosporidium* organisms. Consequently, we developed a predictive model of weekly cryptosporidiosis cases using weekly incidence data (1989–1999) and national data on temperature, rainfall, river discharges, and reported number of travel-associated cases. Separate models were produced for different periods of the year. Ordinary least-squares regression was used for analyses.

The results indicated that between mid-March and the end of June cryptosporidiosis cases were positively associated with river discharges that occurred 2 weeks previously. From July through early September, cryptosporidiosis was positively associated with warm, dry weather in the previous 2 months. No associations between cryptosporidiosis and weather existed at other times. Travel cases were not significant in any of the models. The detailed methods and results of this analysis are available from the author. The results are consistent with previous research (*4*,*5*).

Comparable data on temperature, rainfall, and river discharges were obtained for the postregulation period (2000–2005) and entered into the predictive model. This estimated the number of cases that would have been expected, for each week, from 2000 through 2005. To provide an overview of these predictions, the estimates were summed to produce totals for each half of the year, for every year after regulation.

The results are presented in the Table alongside the 95% confidence interval of the prediction, the actual numbers of cases reported, and the difference between the actual and predicted cases. In the first half of the year, cryptosporidiosis was significantly reduced (p<0.05) every year since 2000. For this finding to be attributable to the regulations, other factors important in cryptosporidiosis etiology should have remained constant during this period. Cryptosporidiosis has been associated with recreational swimming and person-to-person contact and, to our knowledge, the levels of these have remained unchanged.

**Table:** Predicted and observed cryptosporidiosis England and Wales, 2000 to mid-2005

Period		Predicted cases	95% Confidence interval	Observed cases	Predicted minus observed	Reported travel-associated cases
First half of year (weeks 1–26)	2000	2,431	2,161–2,699	1,890	541	85
	2001	2,510	2,242–2,776	925	1,585	54
	2002	2,200	1,932–2,467	1,103	1,097	47
	2003	2,107	1,840–2,373	1,150	957	41
	2004	2,159	1,892–2,425	1,316	843	42
	2005	2,021	1,744–2,297	931	1,090	30
Second half of year (weeks 27–52)	2000	2,438	2,140–2,734	3,477	−1,039	322
	2001	2,927	2,627–3,226	2,461	466	194
	2002	2,294	1,996–2,591	1,795	499	65
	2003	2,713	241–3,010	4,287	−1,574	366
	2004	2,552	2,241–2,863	2,198	354	69

The greatest reduction in cases occurred in the first half of 2001, a period that coincides with the foot-and-mouth disease epidemic. This epidemic led to the slaughter of >6 million livestock and restricted public access to agricultural land (*6*). The large reduction in cases in 2001 has been attributed to this epidemic (*7*,*8*), but our results indicate that cases were already depressed in the first half of 2000, and these reductions continued into 2002. Therefore, the large reduction observed in the first half of 2001 is also likely to be due to the new drinking water regulations.

Another reason for lower cryptosporidiosis incidence since 2000 could be lower levels of *Cryptosporidium* spp. in livestock after the foot-and-mouth epidemic (*9*). However, a recent study has discounted this (*3*), and factors associated with the 2001 epidemic cannot explain the reductions in cases observed in 2000. We conclude, therefore, that improved water treatment associated with the new drinking water regulations has led to cryptosporidiosis reductions during the first half of the year.

In the second half of the year, the pattern is less straightforward. The numbers of cases are significantly (p<0.05) lower than predicted in 2001, 2002, and 2004, but significantly higher (p<0.05) in 2000 and 2003. One explanation for the excess cases in the second half of 2000 and 2003 is that they may represent unreported travel-associated cases or community transmission from these cases. The Table demonstrates that many foreign travel–associated cases occurred in both these periods (>300 in 2000 and 2003 compared with <200 for other years), and these are poorly recorded in national surveillance (*10*). This inconsistency in the pattern between years, combined with the potential link between excess cases and travel-associated cases, led us to conclude that the overall increase in incidence in the second half of the year is not likely to be related to the regulations.

## Conclusions

By averaging the differences between the observed and predicted cryptosporidiosis cases across the years, we can estimate the public health benefits of the regulations. The average excludes 2001 because of the confounding effect of the foot-and-mouth epidemic. Since 2000, an annual average reduction of 615 reported cases has occurred. This reduction comprises a large decrease in the first half of the year and a small increase in the second half. If we assume that the increase in cases in the second half of the year is not associated with drinking water, the benefit of the intervention is 905 reported cases per year (the average reduction in the first half of the year).

Not all cases of cryptosporidiosis in the community are reported to national surveillance, and the ratio of reported to community cases is estimated to be 7.4 (*11*). This multiplier has uncertainties because it is based upon a single study. If this multiplier is applied to our estimate of 905 cases, it implies 6,770 fewer cases of cryptosporidiosis in the community each year. Two recent reports have suggested that even this multiplier may be an underestimate (*12*,*13*).

We have presented evidence that new drinking water regulations implemented in England and Wales during 2000 led to significantly fewer cryptosporidiosis cases in the first half of the year with no significant change in the second half of the year. We estimate a reduction in reported cases of 905 per year or ≈6,770 cases in the community each year. These findings indicate that regulations such as those implemented in England and Wales can have a significant public health benefit in reducing cases of cryptosporidiosis.
